# Case report: Two unique nonsense mutations in *HTRA1*-related cerebral small vessel disease in a Chinese population and literature review

**DOI:** 10.3389/fneur.2022.1069453

**Published:** 2022-12-22

**Authors:** Weijie Chen, Yuanyuan Wang, Shengwen Huang, Xiaoli Yang, Liwei Shen, Danhong Wu

**Affiliations:** Department of Neurology, Shanghai Fifth People's Hospital, Fudan University, Shanghai, China

**Keywords:** *HTRA1*, cerebral small vessel disease, nonsense, ischemic stroke, heterozygous mutations

## Abstract

**Background:**

Homozygous or compound heterozygous mutations in the high-temperature requirement A serine protease 1 gene (*HTRA1*) elicits cerebral autosomal recessive arteriopathy with subcortical infarcts and white matter lesions (CARASIL). The relationship between some heterozygous mutations, most of which are missense ones, and the occurrence of cerebral small vessel diseases (CSVD) has been reported. Recently, heterozygous *HTRA1* nonsense mutations have been recognized to be pathogenic.

**Case presentation:**

We described two Chinese patients diagnosed with *HTRA1*-CSVD accompanied by heterozygous nonsense mutations. Their first clinical manifestations were symptoms due to ischemic stroke, and brain Magnetic Resonance Imaging (MRI) showed diffuse white matter lesions (WMLs) and microbleeds in both of them. Genetic sequencing revealed two novel heterozygous nonsense mutations: c.1096G>T (p.E366X) and c.151G>T (p.E51X).

**Conclusion:**

This case report expands the clinical, radiographic, and genetic spectrum of *HTRA1*-CSVD. Attention should be paid to young patients with ischemic stroke as the first clinical manifestation. Genetic screening for such sporadic CSVD is recommended, even if the symptoms are atypical.

## Introduction

Cerebral small vessel diseases (CSVD) are a series of clinical, imaging, and pathological syndromes that affect small arteries, arterioles, venules, and capillaries within the brain. It increases the risk of acute cerebrovascular events and mainly manifests in cognitive dysfunction (1). Cerebral autosomal recessive arteriopathy with subcortical infarcts and white matter lesions (CARASIL) is a monogenic CSVD, determined by mutations in the high-temperature requirement A serine protease 1 gene (*HTRA1*) which is located on chromosome 10 (10q26) and encodes the high-temperature requirement serine protease.

*HTRA1* mutations include heterozygous, homozygous, and compound heterozygous ones. There are two possible phenotypes related to *HTRA1* mutations: classic CARASIL (OMIM 600142) and CADASIL2 which is also known as *HTRA1*-CSVD (OMIM 616779) (2). Classic CARASIL is caused by homozygous or compound heterozygous mutations inherited in an autosomal recessive manner. *HTRA1*-CSVD is caused by heterozygous mutations inherited in an autosomal dominant pattern (3). Both of them are clinically characterized by progressive gait impairment, acute ischemic stroke at a young age, and cognitive decline, often accompanied by extra-neurological symptoms, such as alopecia and lumbago associated with spondylosis deformans. Compared to classic CARASIL, *HTRA1*-CSVD is generally milder with a lower incidence of extra-neurological symptoms, such as alopecia and lumbago, and neurological symptoms present later (4). Neuroimaging features (severe leukoencephalopathy, lacunar infarctions, and microbleeds) are seen in both classic CARASIL and *HTRA1*-CSVD, but the latter tends to have milder changes (5).

Studies have reported that different mutation sites or types can lead to loss of or decrease in activities of the *HTRA1* serine protease through different mechanisms (6–8). The pathogenicity of heterozygous pathogenic missense variants mainly results from a significant decrease in the activities of the protease and a dominant-negative effect on the trimer (6, 7). However, the underlying molecular mechanism of heterozygous mutations is not fully understood.

Recent studies have found that nonsense mutations are also pathogenic. Thus, there are 11 heterozygous nonsense mutations according to the ClinVar database, but clinical features and the possible correlation between genotypes and phenotypes of *HTRA1*-CSVD are barely understood. In this case report, we presented two patients with ischemic stroke at a young age, which was related to novel nonsense mutations. Ischemic stroke was the first clinical manifestation, and brain MRI of these patients showed typical white matter lesions (WMLs) and microbleeds. Genetic sequencing revealed two novel heterozygous nonsense mutations of c.1096G>T (p.E366X) and c.151G>T (p.E51X), supporting *HTRA1*-CSVD. Our findings provide further insights into the relationship between genotypes and phenotypes.

## Materials and methods

### Case 1

A 44-year-old man presented to our department due to weakness of the left upper limb over the past 4 days. He had no prevailing risk factors for cerebral infarction such as hypertension, diabetes mellitus, atrial fibrillation, or smoking. He denied any memory loss, dysarthria, or loss of consciousness. He had no history of alopecia or lumbago. None of his family members had relevant health problems or hereditary diseases.

**Figure 1:**
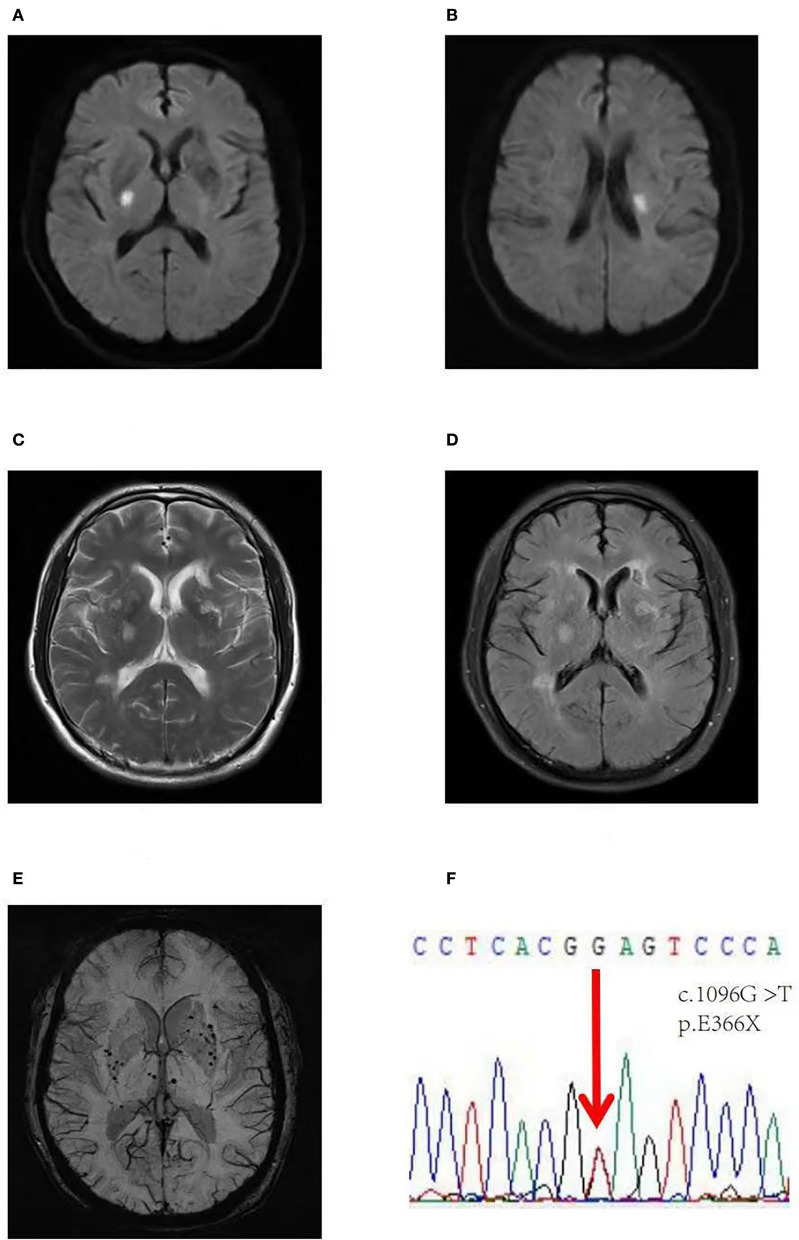
Brain axial images of the first *HTRA1*-CSVD patient. A DWI image showing an acute infarct in the right basal ganglia **(A)**, and the second acute infarct in the region next to the left lateral ventricle **(B)**, axial T2-weighted **(C)**, and FLAIR axial images **(D)** showing diffuse WMLs, Fazekas scale score 2, lacunar lesions in bilateral regions next to the lateral ventricle, and an SWI image **(E)** showing chronic microbleeds in the deep white matter. **(F)** Validation of a heterozygous G to T mutation at position 1,096 (NM_002775: c.1096G>T) in exon 6 of the *HTRA1* gene, resulting in a stop codon at 366 (p.E366X). FLAIR, fluid attenuated inversion recovery; MRI, magnetic resonance imaging; DWI, diffusion-weighted imaging; SWI, susceptibility-weighted imaging.

Neuropsychological tests showed no signs of cognitive impairment. His blood indicators, cervical vascular ultrasound, and dynamic electrocardiogram showed no significant abnormalities. Brain MRI showed an acute infarct in the right basal ganglia on DWI images (Figure 1A), diffuse WMLs, Fazekas scale score 2, bilateral lacunar lesions in regions close to the lateral ventricle on T2W/FLAIR, and chronic microbleeds in the deep white matter on SWI images.

During the follow-up, this patient was admitted to the hospital because of recurrent ischemic stroke despite antiplatelet therapy with no complaint about cognitive impairment. Brain MRI showed acute infarct in the region next to the left lateral ventricle on DWI (Figure 1B), diffuse WMLs and chronic microbleeds were the same as previously reported.

Prompted by the unusual clinical phenotype and neuroimaging findings, a hereditary form of CSVD was suspected (Figures 1C–E). Whole-exome sequencing identified a novel heterozygous *HTRA1* nonsense mutation (NM_002775: C.1096G>T; p.E366X) (Figure 1F). The variant had not been previously reported in the scientific literature or the HGMD and was not found in reference population databases such as gnomAD, ExAC, or 1000 Genome Project. The Mutation-Taster score was 1. The proband's parents had passed away, and neither the proband's brother nor his son showed abnormality in the proband's mutation site upon gene testing. Based on the clinical manifestations of this patient and gene sequencing results, which are concordant with each other, this variant has been proposed to be pathogenic.

### Case 2

A 53-year-old man presented to our department due to dysarthria and weakness of the left limb for 17 hours. He had no past medical history and vascular risk factors. The patient could not perform some simple tasks in daily life and lost his previous interests at the age of 49. He did not have migraine, alopecia, or lumbago, which are characteristic features of monogenic CSVD. His family history was unremarkable and his family members were all healthy.

On neurological examination, the patient showed a pronounced slow response to cognitive tests. The Mini-Mental State Examination (MMSE) score was 24 and the Montreal Cognitive Assessment (MoCA) score was 17. His cerebral vessels showed no significant abnormalities on cerebrovascular examination. Brain MRI showed acute infarct of the left corona radiata on DWI images, diffuse WMLs and brain atrophy, Fazekas scale score 3 on T2W/FLAIR images, chronic microbleeds in the lobar regions, and deep white matter on SWI images.

**Figure 2:**
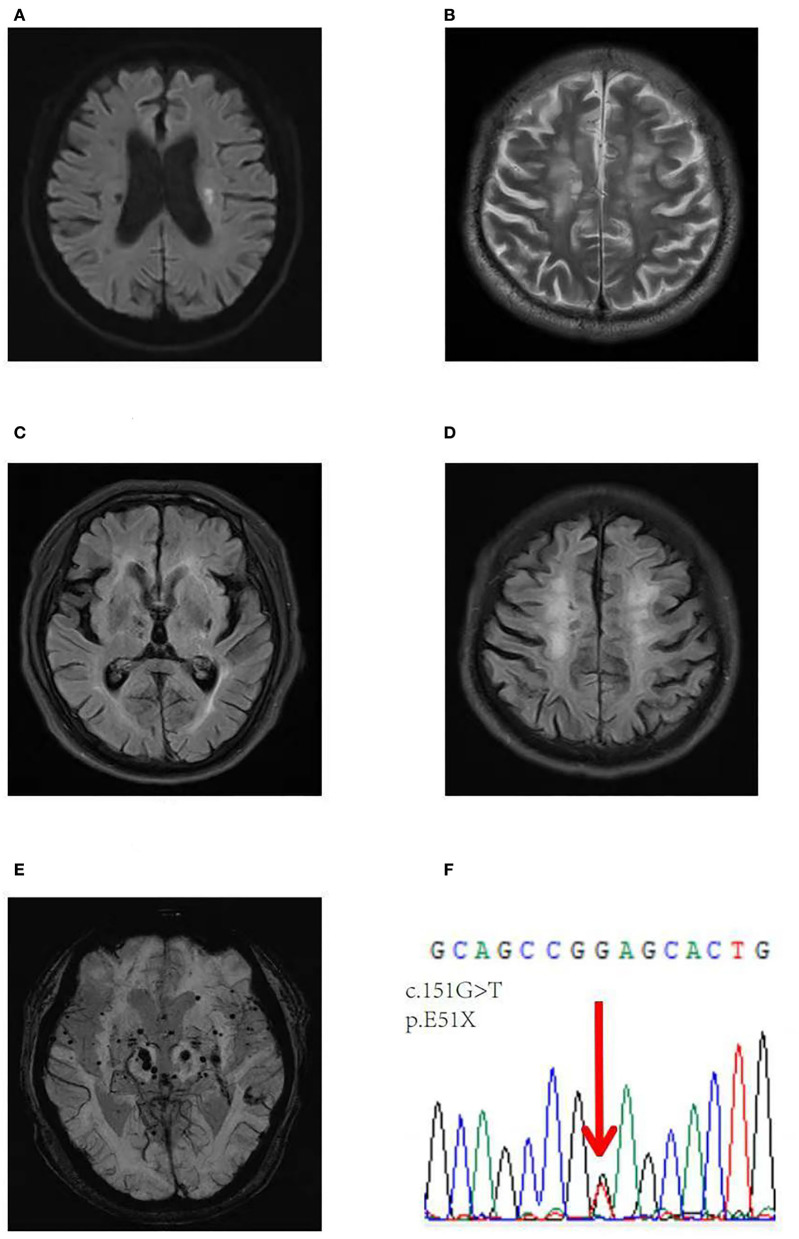
Brain axial images of the second patient. A DWI image showing an acute infarct in the left corona radiata **(A)**, axial T2-weighted **(B)** and FLAIR axial images **(C, D)** showing diffuse WMLs and brain atrophy, Fazekas scale score 3, and an SWI image showing chronic microbleeds in lobar regions and the deep white matter **(E)**. **(F)** Validation of a heterozygous G to T mutation at position 151 (NM_002775: c.151G>T) in exon 1 of the *HTRA1* gene, resulting in a stop codon at 51 (p.E51X).

Given the presentation of early-onset progressive dementia, ischemic stroke at a young age, and brain MRI demonstrating extensive WMLs and brain atrophy (Figures 2A–E), whole-exome sequencing was performed. Whole-exome sequencing identified a novel heterozygous nonsense mutation (NM_002775: c.151G>T; p.E51X) (Figure 2F). This variant has not been previously reported in the scientific literature or the HGMD and was not found in reference population databases, such as gnomAD, ExAC, or 1000 Genome Project, and the Mutation-Taster score was 1. This variant has been classified as pathogenic according to the ACMG pathogenicity rating.

## Discussion

In the present study, we reported two *HTRA1*-CSVD patients with ischemic stroke at a young age, with two unique nonsense mutations: c.1096G >T (p.E366X) and c.151G>T (p.E51X). To date, only 11 heterozygous nonsense mutation sites have been reported. Coste et al. (9) have found that the heterozygous *HTRA1* stop codon variants are not restricted to a specific domain but are present throughout the gene. The present p.E366X and p.E51X mutations may lead to nonsense-mediated mRNA decay (NMD) or truncation of the protein in front of the functional protease domain. These conditions may result in the loss of protein functions. Coste et al. (9) have found that heterozygous *HTRA1* stop codon variants are dominant with an age-dependent and incomplete clinical penetrance in the analysis of 3,336 patients with CSVD without known pathogenic mutations. We hypothesize that this might be the reason why family members of the patients in our study did not have relevant health problems. Therefore, careful follow-up was warranted for their relatives.

The clinical and radiographic spectra of these two patients were similar to those in other studies. Compared with classic CARASIL, heterozygous *HTRA1* pathogenic variants may have a lower incidence of extra-neurological symptoms and manifest neurological symptoms later (4). Onodera et al. have found that acute ischemic stroke usually occurs after 40 years, which is consistent with the results of the patients in our study. In addition, ischemic stroke was the first clinical manifestation in our study. Coste et al. have drawn a similar conclusion when conducting clinical and imaging analyses of 11 symptomatic carriers of nonsense mutations. Stroke or transient ischemic attack (TIA) is the first clinical manifestation in seven probands (64%) (9). Therefore, ischemic stroke is the essential clinical manifestation of CSVD. Patients with ischemic stroke at a young age need particular attention due to the high likelihood of being a carrier of *HTRA1* mutations. Neither of the two patients in our study had apparent symptoms of alopecia or lumbago, which was consistent with previous findings, in which *HTRA1*-CSVD had a lower incidence of extra-neurological symptoms than classic CARASIL (10).

Radiologic hallmarks of CARASIL include high-signal-intensity lesions in the periventricular and deep white matter and multiple lacunar infarcts in the basal ganglia and the thalamus, but the superficial white matter is generally spared (11). Whittaker et al. (12) have also reported that microbleeds are mostly found in the deep white matter of the brain in patients with heterozygous *HTRA1* mutations. Our findings are consistent with the results of the above studies, in that both of these patients had diffuse WMLs and chronic microbleeds in the deep white matter and lacunar infarcts in the basal ganglia and the corona radiata.

Pathogenic mechanisms of *HTRA1*-CSVD are gaining popularity in recent years. Different types of mutations may lead to discrete protease activities. Heterozygous missense mutations take a dominant-negative effect on the activities of the protease. Nozaki et al. have demonstrated that heterozygous mutations may fail to form stable trimers and interrupt the *HTRA1* activation cascade, resulting in a dominant-negative effect on the wild-type protease (7). Lee et al. (4) have further confirmed that the core feature of *HTRA1*-CSVD may be the disturbance of activities of the protease. However, the detailed molecular mechanisms of heterozygous nonsense mutations are not fully understood. A study proposed that heterozygous nonsense or frameshift variants were pathogenic through a haploinsufficiency mechanism which resulted in the degradation of the mutated mRNA at the location of these variants (9). Further studies are needed to elucidate the pathogenic mechanisms.

## Conclusion

We reported two novel heterozygous nonsense mutations which expand the mutation spectrum of *HTRA1*. The age-dependent and incomplete clinical penetrance of nonsense mutations of *HTRA1* increase the difficulty of gene detection; therefore, a larger number of clinical samples and a longer follow-up are necessary to screen these patients. Meanwhile, attention should be paid to young patients with ischemic stroke as their first clinical manifestation. Genetic screening for such sporadic small vascular disease is recommended, even if the symptoms are atypical.

## Data availability statement

The datasets presented in this article are not readily available because of ethical and privacy restrictions. Requests to access the datasets should be directed to the corresponding author.

## Ethics statement

The studies involving human participants were reviewed and approved by the Ethics Committee of Shanghai Fifth People's Hospital of Fudan University. The patients/participants provided their written informed consent to participate in this study. Written informed consent was obtained from the individual(s) for the publication of any potentially identifiable images or data included in this article.

## Author contributions

Preparing the original draft: WC. Reviewing, editing, supervision, and conceptualization: DW. Genetic data analysis: LS and XY. Clinical data acquisition: YW and SH. All authors have read and agreed to publish this report.
