# Supplementary material for: Case report: Two unique nonsense mutations in HTRA1-related cerebral small vessel disease in a Chinese population and literature review
Source: Front Neurol. 2022 Dec 22;13:1069453. doi: 10.3389/fneur.2022.1069453 (PMC9813394; doi:10.3389/fneur.2022.1069453)
Supplement: Supplementary file 1 [file Table_1.DOCX]

CARE Checklist

1、Title – case report：Two Unique Nonsense Mutations in HTRA1-Related Cerebral Small Vessel Disease in a Chinese Population and Literature Review

2、Key Words – HTRA1; cerebral small vessel disease; nonsense mutation; ischemic stroke; case report

3、Abstract

Introduction

We described two Chinese patients diagnosed with HTRA1-CSVD accompanied by heterozygous nonsense mutations.

Their first clinical manifestations were symptoms due to ischemic stroke.

brain Magnetic Resonance Imaging (MRI) showed diffuse white matter lesions (WMLs) and microbleeds in both of them. Genetic sequencing revealed two novel heterozygous nonsense mutations: c.1096G >T (p.E366X) and c.151G>T (p.E51X).

This case report expands the clinical, radiographic, and genetic spectrum of HTRA1-CSVD. Attention should be paid to young patients with ischemic stroke as the first clinical manifestation. Genetic screening for such sporadic CSVD is recommended, even if the symptoms are atypical.4、Introduction – Recent studies have found that HTRA1 nonsense mutations are also pathogenic. So far, as there are only a little more than 11 symptomatic carriers of HTRA1 pathogenic variants and around 8 mutations distinguished in the literature.

5、Patient Information

Case 1:A 44-year-old man presented to our department due to weakness of the left upper limb over the past 4 days. He had no prevailing risk factors of cerebral infarction such as hypertension, diabetes mellitus, atrial fibrillation, or smoking. He denied any memory loss, dysarthria or loss of consciousness. He had no history of alopecia or lumbago. None of his family members had relevant health problems or hereditary diseases.During the follow-up, this patient was admitted to the hospital because of recurrent ischemic stroke in spite of antiplatelet therapy with no complaint about cognitive impairment. Prompted by the unusual clinical phenotype and neuroimaging findings, a hereditary form of CSVD was suspected, the Whole-exome sequencing identified a novel heterozygous HTRA1 gene (NM_002775: C.1096G >T), the variant had not been previously reported in the scientific literature or the HGMD and was not found in reference population databases gnomAD, ExAC, or 1000 Genome Project, the Mutation-Taster score was 1, the variant has been classified as pathogenic by ACMG.

Case 2: A 53-year-old man presented to our department due to dysarthria and weakness of the left limb for 17 hours. He had no past medical history and vascular risk factors. The patient could not perform some simple tasks in daily life and lost his previous interests since the age of 49. He did not have migraine, alopecia or lumbago, which are characteristic features of monogenic CSVD. His family history was unremarkable and his family members were all healthy. Given the presentation of early onset progressive dementia and ischemic stroke at a young age, and brain MRI demonstrating extensive WMLs and brain atrophy , the Whole-exome sequencing identified a novel heterozygous HTRA1 gene (NM_002775: c.151G>T), the variant had not been previously reported in the scientific literature or the HGMD and was not found in reference population databases gnomAD, ExAC, or 1000 Genome Project, the Mutation-Taster score was 1. the variant has been classified as Uncertain significance according to the ACMG pathogenicity rating.

6、Clinical Findings

Case 1: Brain MRI showed an acute infarct in the right basal ganglia on DWI images (Figure 1A), diffuse WMLs, Fazekas scale score 2, bilateral lacunar lesions in regions close to the lateral ventricle on T2W/FLAIR, and chronic microbleeds in the deep white matter on SWI images. Secondly, brain MRI showed acute infarct in the region next to the left lateral ventricle on DWI .

Case 2: On neurological examination, the patient showed pronounced slow response to cognitive tests. The Mini-Mental State Examination (MMSE) score was 24, and the Montreal Cognitive Assessment (MoCA) score was 17. His cerebral vessels showed no significant abnormalities on cerebrovascular examination. Brain MRI showed acute infarct of the left corona radiata on DWI images, diffuse WMLs and brain atrophy, Fazekas scale score 3 on T2W/FLAIR images, chronic microbleeds in the lobar regions and deep white matter on SWI images. .

7、Diagnostic Assessment

laboratory testing: the Whole-exome sequencing

Imaging:FLAIR: fluid attenuated inversion recovery; MRI: magnetic resonance imaging, DWI: Diffusion Weighted Imaging. SWI: Susceptibility weighted imaging

8、Therapeutic Intervention

Maintenance of current treatment

9、Follow-up and Outcomes

In progress

10、Discussion

Strengths and limitations in your approach to this case.

We described two cases of Chinese patients diagnosed with HTRA1-CSVD companied with heterozygous nonsense mutations. Recent studies have found that nonsense mutations are also pathogenic. So far, There are 11 heterozygous nonsense mutations according to the clinvar database, but clinical features and the possible correlation between genotypes and phenotypes of HTRA1-CSVD are barely understood.

Discussion of the relevant medical literature.

In the present study, we reported two HTRA1-CSVD patients with ischemic stroke at a young age, with two unique nonsense mutations: c.1096G >T (p.E366X) and c.151G>T (p.E51X). To date, only 11 heterozygous nonsense mutation sites have been reported. Coste et al have found that the heterozygous HTRA1 stop codon variants are not restricted to a specific domain but present throughout the gene [9]. The present p.E366X and p.E51X mutations may lead to nonsense-mediated mRNA decay (NMD) or to truncation of the protein in front of the functional protease domain. These conditions may result in loss of protein functions. Coste et al have found that heterozygous HTRA1 stop codon variants are dominant with an age-dependent and incomplete clinical penetrance in the analysis of 3,336 CSVD patients without known pathogenic mutations [9]. We hypothesize that this might be the reason why family members of the patients in our study did not have relevant health problems. Therefore, careful follow-up was warranted for their relatives.

The clinical and radiographic spectra of these two patients were similar to those in other studies. Compared with classic CARASIL, heterozygous HTRA1 pathogenic variants may have a lower incidence of extra-neurological symptoms and manifest neurological symptoms later [4]. Onodera et al have found that acute ischemic stroke usually occurs after 40 years, which is consistent with the results of the patients in our study. In addition, ischemic stroke was the first clinical manifestation in our study. Coste et al have drawn a similar conclusion when conducting clinical and imaging analyses of 11 symptomatic carriers of nonsense mutations. Stroke or transient ischemic attack (TIA) is the first clinical manifestation in 7 probands (64%)[9]. Therefore, ischemic stroke is the essential clinical manifestation of CSVD. Patients with ischemic stroke at a young age need particular attention due to the high likelihood of being a carrier of HTRA1 mutations. Neither of the two patients in our study had apparent symptoms of alopecia or lumbago, which was consistent with previous findings that HTRA1-CSVD had a lower incidence of extra-neurological symptoms than classic CARASIL [10].

Radiologic hallmarks of CARASIL include high-signal-intensity lesions in the periventricular and deep white matter, multiple lacunar infarcts in the basal ganglia and the thalamus, but superficial white matter is generally spared [11]. Whittaker et al have also reported that microbleeds are mostly found in the deep white matter of the brain in patients with heterozygous HTRA1 mutations [12]. Our findings are consistent with results of the above studies in that both of these patients had diffuse WMLs and chronic microbleeds in the deep white matter, and lacunar infarcts in the basal ganglia and the radiographic corona.

The rationale for your conclusions.

Firstly, the clinical and radiographic spectrum of the two patients were similar to those of other HTRA1-CSVD, we had the same radiologic hallmarks as other studies.

Secondly, we reported two novel heterozygous nonsense mutations in HTRA1, the variant had not been previously reported in the scientific literature or the HGMD and was not found in reference population databases gnomAD, ExAC, or 1000 Genome Project, the Mutation-Taster score was 1.The variants have been classified as pathogenic according to the ACMG pathogenicity rating.

Thirdly, The present p.E366X and p.E51X mutations meet the criteria of the nonsense-mediated mRNA decay and, therefore, lead to haploinsufficiency. We concluded that the heterozygous p.E366X and p.E51X mutations were sufficient to result in CSVD. Coste et al have found that heterozygous HTRA1 stop codon variants are dominant with an age-dependent and incomplete clinical penetrance in the analysis of 3,336 CSVD patients without known pathogenic mutations. We hypothesize that this might be the reason why family members of the patients in our study did not have relevant health problems. Therefore, careful follow-up was warranted for their relatives.

The primary “take-away” lessons from this case report (without references) in a one paragraph 11、Conclusion.

We reported two novel heterozygous nonsense mutations which expand the mutation spectrum of HTRA1. The age-dependent and incomplete clinical penetrance of nonsense mutations of HTRA1 increases the difficulty of gene detection, therefore, a larger number of clinical samples and a longer follow-up are necessary to screen these patients. Meanwhile, attention should be paid to young patients with ischemic stroke as their first clinical manifestation. Genetic screening for such sporadic small vascular disease is recommended, even if the symptoms are atypical.

1. Patient Perspective – The patients supported the treatment(s) they received.

13、Informed Consent – The patients gave informed consent.
