# Supplementary material for: Case report: Two unique nonsense mutations in HTRA1-related cerebral small vessel disease in a Chinese population and literature review
Source: Front Neurol. 2022 Dec 22;13:1069453. doi: 10.3389/fneur.2022.1069453 (PMC9813394; doi:10.3389/fneur.2022.1069453)
Supplement: Supplementary file 2 [file Data_Sheet_1.ZIP › case-A/1.测序统计/Quality/Raw/CA2245B_R1_fastqc.html]

CA2245B\_R1.fq.gz FastQC Report 

FastQC Report

Wed 21 Jul 2021  
CA2245B\_R1.fq.gz

## Summary

- Basic Statistics
- Per base sequence quality
- Per tile sequence quality
- Per sequence quality scores
- Per base sequence content
- Per sequence GC content
- Per base N content
- Sequence Length Distribution
- Sequence Duplication Levels
- Overrepresented sequences
- Adapter Content

## Basic Statistics

| Measure | Value |
| --- | --- |
| Filename | CA2245B\_R1.fq.gz |
| File type | Conventional base calls |
| Encoding | Sanger / Illumina 1.9 |
| Total Sequences | 58473352 |
| Sequences flagged as poor quality | 0 |
| Sequence length | 150 |
| %GC | 53 |

## Per base sequence quality

## Per tile sequence quality

## Per sequence quality scores

## Per base sequence content

## Per sequence GC content

## Per base N content

## Sequence Length Distribution

## Sequence Duplication Levels

## Overrepresented sequences

No overrepresented sequences

## Adapter Content

Produced by FastQC (version 0.11.8)
