# Supplementary material for: Case report: Two unique nonsense mutations in HTRA1-related cerebral small vessel disease in a Chinese population and literature review
Source: Front Neurol. 2022 Dec 22;13:1069453. doi: 10.3389/fneur.2022.1069453 (PMC9813394; doi:10.3389/fneur.2022.1069453)
Supplement: Supplementary file 5 [file Data_Sheet_4.ZIP › case2-A/1.测序统计/Quality/Clean/CA2275B.rmadapt.1_fastqc.html]

CA2275B.rmadapt.1.fq FastQC Report 

FastQC Report

Wed 11 Aug 2021  
CA2275B.rmadapt.1.fq

## Summary

- Basic Statistics
- Per base sequence quality
- Per tile sequence quality
- Per sequence quality scores
- Per base sequence content
- Per sequence GC content
- Per base N content
- Sequence Length Distribution
- Sequence Duplication Levels
- Overrepresented sequences
- Adapter Content

## Basic Statistics

| Measure | Value |
| --- | --- |
| Filename | CA2275B.rmadapt.1.fq |
| File type | Conventional base calls |
| Encoding | Sanger / Illumina 1.9 |
| Total Sequences | 56938831 |
| Sequences flagged as poor quality | 0 |
| Sequence length | 10-150 |
| %GC | 50 |

## Per base sequence quality

## Per tile sequence quality

## Per sequence quality scores

## Per base sequence content

## Per sequence GC content

## Per base N content

## Sequence Length Distribution

## Sequence Duplication Levels

## Overrepresented sequences

No overrepresented sequences

## Adapter Content

Produced by FastQC (version 0.11.8)
